# OA12.01. Analysis of FDA mandated dietary supplement adverse event reports (AER) 2008-2009

**DOI:** 10.1186/1472-6882-12-S1-O45

**Published:** 2012-06-12

**Authors:** M Hardy, S Stumpf, M Zimmerman, C Carr

**Affiliations:** 1 Simms/Mann-UCLA Center for Integrative Oncology, Los Angeles, USA; 2 American Herbal Products Association, Washington D.C., USA

## Purpose

Safety of dietary supplements has been an area of concern for regulators, the health care community and consumers. The ability of the FDA to assess validity of these concerns has been hampered by small numbers of voluntary AE reports. In 2006 Congress mandated all serious adverse events be reported by dietary supplement manufacturers. We present the first systematic analysis of the initial two years of reports.

## Methods

We obtained AER’s for 2008 and 2009 from the FDA (n=2,288). Data were abstracted by a single reviewer, confirmed by at least one other, entered into a standard excel sheet, and analyzed using SPSS. Non-parametric tests of significance were also applied.

## Results

In preliminary results, manufacturers accounted for 57.8% of all reports, consumers 29.6% and health care professionals 9.7%. 61.8% of subjects were female. The most commonly reported dietary ingredients were combination products (33.5%), vitamin/mineral (19%), and plant or isolated plant constituent (15.8%). 297 reports included multiple products. Fifty-five percent of reports included the label or label information. The most common adverse outcome was hospitalization (750, 32.8%). Thirty-one deaths were reported (1.4% total reports), 56 episodes of disability (2.4%), 233 life-threatening complications (10.2%), and 943 other serious adverse events (41.2%). A detailed assessment of deaths, hospitalizations and life threatening complications will be presented. No single product type or ingredient was significantly associated with death. Attempts to apply the WHO-UMC causality assessment system yielded limited results and would be improved with the collection of more sufficient data. A cluster of serious adverse events involving a single exercise enhancement product was identified.

## Conclusion

Mandated reporting has increased manufacturer reports but missing data limits analysis. Preliminary results do not suggest a wide spread problem with dietary supplement safety. This analysis did identify a cluster of cases representing a potential public health concern. Directions for future research will be discussed.

